# Nkx2.5 Functions as a Conditional Tumor Suppressor Gene in Colorectal Cancer Cells *via* Acting as a Transcriptional Coactivator in p53-Mediated p21 Expression

**DOI:** 10.3389/fonc.2021.648045

**Published:** 2021-04-01

**Authors:** Huili Li, Jiliang Wang, Kun Huang, Tao Zhang, Lu Gao, Sai Yang, Wangyang Yi, Yanfeng Niu, Hongli Liu, Zheng Wang, Guobin Wang, Kaixiong Tao, Lin Wang, Kailin Cai

**Affiliations:** ^1^ Department of Gastrointestinal Surgery, Union Hospital, Tongji Medical College, Huazhong University of Science and Technology, Wuhan, China; ^2^ Institution of Cardiology, Union Hospital, Tongji Medical College, Huazhong University of Science and Technology, Wuhan, China; ^3^ Department of Anesthesiology, Union Hospital, Tongji Medical College, Huazhong University of Science and Technology, Wuhan, China; ^4^ Department of Cardiology, The First Affiliated Hospital of Zhengzhou University, Zhengzhou, China; ^5^ Department of General Surgery, The Second People’s Hospital of Jingmen, Jingmen, China; ^6^ Cancer Center, Union Hospital, Tongji Medical College, Huazhong University of Science and Technology, Wuhan, China; ^7^ Research Center for Tissue Engineering and Regenerative Medicine, Union Hospital, Tongji Medical College, Huazhong University of Science and Technology, Wuhan, China; ^8^ Department of Clinical Laboratory, Union Hospital, Tongji Medical College, Huazhong University of Science and Technology, Wuhan, China

**Keywords:** Nkx2.5 gene, colorectal cancer, tumor suppressor gene, TP53 gene mutation, p21^WAF1/CIP1^

## Abstract

NK2 homeobox 5 (Nkx2.5), a homeobox-containing transcription factor, is associated with a spectrum of congenital heart diseases. Recently, Nkx2.5 was also found to be differentially expressed in several kinds of tumors. In colorectal cancer (CRC) tissue and cells, hypermethylation of Nkx2.5 was observed. However, the roles of Nkx2.5 in CRC cells have not been fully elucidated. In the present study, we assessed the relationship between Nkx2.5 and CRC by analyzing the expression pattern of Nkx2.5 in CRC samples and the adjacent normal colonic mucosa (NCM) samples, as well as in CRC cell lines. We found higher expression of Nkx2.5 in CRC compared with NCM samples. CRC cell lines with poorer differentiation also had higher expression of Nkx2.5. Although this expression pattern makes Nkx2.5 seem like an oncogene, *in vitro* and *in vivo* tumor suppressive effects of Nkx2.5 were detected in HCT116 cells by establishing Nkx2.5-overexpressed CRC cells. However, Nkx2.5 overexpression was incapacitated in SW480 cells. To further assess the mechanism, different expression levels and mutational status of p53 were observed in HCT116 and SW480 cells. The expression of p21^WAF1/CIP1^, a downstream antitumor effector of p53, in CRC cells depends on both expression level and mutational status of p53. Overexpressed Nkx2.5 could elevate the expression of p21^WAF1/CIP1^ only in CRC cells with wild-type p53 (HCT116), rather than in CRC cells with mutated p53 (SW480). Mechanistically, Nkx2.5 could interact with p53 and increase the transcription of p21^WAF1/CIP1^ without affecting the expression of p53. In conclusion, our findings demonstrate that Nkx2.5 could act as a conditional tumor suppressor gene in CRC cells with respect to the mutational status of p53. The tumor suppressive effect of Nkx2.5 could be mediated by its role as a transcriptional coactivator in wild-type p53-mediated p21^WAF1/CIP1^ expression.

## Introduction

Colorectal cancer (CRC) is one of the most common malignancies with high rates of morbidity and mortality (1–4). The formation of CRC is a multistep process that arises from accumulation of genetic and epigenetic alterations (5, 6), including silencing of tumor suppressor genes with aberrant methylations, as well as activating oncogenes by mutations and/or chromosomal deletions (7). This has led to the hypothesis that aberrant methylation could be used as a marker to identify candidate tumor suppressor genes in neoplasia (7, 8). The CpG islands hypermethylation of homeobox transcriptional factor NK2 homeobox 5 (Nkx2.5, also known as CSX) has been reported in previous CRC-based studies (9, 10). Thus, Nkx2.5 was considered as a candidate tumor suppressor for CRC (9, 10).

Nkx2.5 is a homeobox-containing transcription factor that belongs to the NK2 class of homeobox proteins (11, 12). It is associated with a spectrum of congenital heart diseases (13–16). To date, NK2 homeobox members have been successively reported as tumor suppressors in various tumors (17–26). Nkx2.5 has also been reported to be expressed in several types of tumors (27–30), but its role in these tumors remains undefined. Though the mechanism of how NK2 homeobox members play roles in tumors has still remained elusive, Nkx2.1 has been reported to mediate p53-induced tumor suppression (17–19, 25), which gives us a hint. Nkx2.5 has also been reported to regulate cell proliferation *via* interacting with cell cycle-related pathways (31–35). Along with these clues, Nkx2.5 may act as a tumor suppressor in CRC through making an interaction with a p53 and/or cell cycle-related pathway.

To date, the tumor suppressor protein p53 (encoded by TP53 gene) is the most extensively studied tumor suppressor (36, 37). P53 is one of the central components in the tumor suppressive network (38–40). CDKN1A gene, whose major transcriptional activator is p53 (38–40), encodes p21^WAF1/CIP1^, a protein preventing cell cycle progression (41). P21^WAF1/CIP1^ has been shown to play a critical role in p53-mediated tumor suppression (42–44). A great number of molecules were found to play roles in tumors *via* p53/p21^WAF1/CIP1^ pathway (42, 45–47). Studies also indicated that Nkx2.5 could influence p53-related pathways in cardiomyocyte, fibroblast, and myoblast (33, 48). Although these clues indicate the relationship between Nkx2.5 and p53/p21^WAF1/CIP1^ pathway, the role of Nkx2.5 and its interaction with p53/p21^WAF1/CIP1^ pathway in CRC still needs to be further elucidated.

In the present study, we assessed the role of Nkx2.5 in CRC by comparing the expression of Nkx2.5 in CRC and adjacent normal colonic mucosa (NCM) samples, as well as in CRC cell lines. In addition, we attempted to determine the effect of Nkx2.5 overexpression on behaviors of CRC cells *in vitro* and *in vivo*. Nkx2.5 overexpression leads to different manifestations in different CRC cell lines because of different mutational statuses of p53. It is also noteworthy that Nkx2.5 could interact with p53 to activate p21^WAF1/CIP1^ transcription. This co-activatory effect depends on the mutational status of p53. Taken together, our results suggest that Nkx2.5 functions as a conditional tumor suppressor gene in CRC cells *via* activating the p53-mediated p21^WAF1/CIP1^ expression.

## Materials and Methods

### Ethics Statement

The study was conducted according to ethical standards, the Declaration of Helsinki, and national and international guidelines, and it was approved by the Institutional Review Board of Union Hospital, Tongji Medical College, Huazhong University of Science and Technology (China).

### Data Collection

The graphs of Nkx2.5 expression profiles in CRC and adjacent NCM samples were generated and downloaded from Oncomine microarray database (https://www.oncomine.org).

### Patients’ Samples

Here, 14 pairs of primary human CRC samples and their matched adjacent NCM samples were obtained from CRC patients who were admitted to our hospital and underwent surgical resection in 2015 (see **Supplementary Table 1**). Samples were collected according to the protocol approved by the Institutional Review Board. The patients signed the written informed consent form prior to commencing the study. All the enrolled individuals were Han Chinese. The data were analyzed anonymously.

### Cell Lines

Human FHC, Hela, Caco-2, DLD-1, HCT116, HT-29, SW480, RKO, SW620, LoVo, HEK-293T cells, and H9c2 cells were cultured in a medium recommended by the American Type Culture Collection (ATCC; Manassas, VA, USA) that was supplemented with 10% fetal bovine serum (FBS; HyClone, Logan, UT, USA) and 1% penicillin-streptomycin (Gibco, Carlsbad, CA, USA) at 37°C in a 5% CO2 incubator (Thermo Fisher Scientific, Waltham, MA, USA). Data related to the original tumors or xenografts of CRC cell lines (49) are listed in **Supplementary Table 2**. The identities of the cancer cell lines were confirmed by short tandem repeat (STR) profiling.

### Construction of Plasmids

The Nkx2.5 overexpression plasmid was generated by cloning the full-length of cDNA representing the complete ORF of Nkx2.5 into the pSi-Flag vector (Invitrogen, Carlsbad, CA, USA). Nkx2.5-F (5’-ATGGATCCAATGTTCCCCAGCCCTGCTCT-3’) and Nkx2.5-R (5’-TGCTACCAGGCTCGGATACCATGCAGCGT-3’) were chosen as primers.

### Generation of Infectious Virus

Lentiviral vectors were transfected into HEK-293T cells in combination with the lentiviral packaging vectors (pRSV-Rev, pMD2.G, and pCMV-VSV-G) using Lipofectamine™2000 reagent (Invitrogen, Carlsbad, CA, USA). After transfection for 48 h, supernatants were collected, filtered, and used to establish the Nkx2.5-overexpression cell lines (Lenti-Nkx2.5-HCT116 and Lenti-Nkx2.5-SW480). Empty vectors were used to generate control cell lines (Lenti-NC-HCT116 and Lenti-NC-SW480). Colonies resistant to Geneticin (G418; Invitrogen, Carlsbad, CA, USA) were picked and expanded to obtain stable clone stock cells. Western blot and quantitative reverse transcription-polymerase chain reaction (RT-qPCR) assays were employed to indicate whether stable cell lines could be successfully generated.

### RNA Extraction and RT-qPCR

Total RNA was isolated using TRIzol reagent (Invitrogen, Carlsbad, CA, USA) according to the manufacturer’s instructions. 2 μg of total RNA was reversely transcribed using an RNA PCR kit (Takara Biotechnology, Inc., Otsu, Japan), and resulting cDNA was used as a template for a standard PCR. The mRNA levels were detected by RT-qPCR with an ABI PRISM 7900 Sequence Detector system (Applied Biosystem, Foster City, CA, USA) according to the manufacturer’s instructions, and normalized to glyceraldehyde-3-phosphate dehydrogenase (GAPDH) gene expression. The primers used for RT-qPCR are listed in **Supplementary Table 3** (33).

### Western Blot Assay

Total cells and tissues were lysed using radioimmunoprecipitation assay (RIPA) buffer, and the protein concentration was determined with a bicinchoninic acid (BCA) protein assay kit (Pierce Biotechnology Inc., Rockford, IL, USA). Protein extracts were separated by sodium dodecyl sulfate-polyacrylamide gel electrophoresis (SDS-PAGE; Invitrogen, Carlsbad, CA, USA), and were then transferred onto a nitrocellulose membrane (Millipore, Billerica, MA, USA), which was incubated with various primary antibodies overnight at 4°C. After incubation with horseradish peroxidase (HRP)-conjugated secondary antibodies (dilution, 1:5000) for 1 h at room temperature, the membranes were treated with normal enhanced chemiluminescence (ECL) substrate (170-5061; Bio-Rad Laboratories Inc., Hercules, CA, USA) prior to visualization using a ChemiDoc MP imaging analysis system (Bio-Rad Laboratories Inc., Hercules, CA, USA) according to the manufacturer’s instructions, as previously described (50). The specific protein levels were normalized to GAPDH on the same nitrocellulose membrane. The following primary antibodies were used: anti-Nkx2.5 (sc-376565X; dilution, 1:500; Santa Cruz Biotechnology, Dallas, TX, USA), anti-GAPDH (sc-32233; dilution, 1:1000; Santa Cruz Biotechnology, Dallas, TX, USA), anti-p53 (#2527; dilution, 1:1000; Cell Signaling Technology, Danvers, MA, USA), and anti-p21^WAF1/CIP1^ (#2947; dilution, 1:1000; Cell Signaling Technology, Danvers, MA, USA).

### Colony Formation and Cell Proliferation Assays

For colony formation, 1000 cells/well were seeded into 6-well plates. After 14 days of culture, cells were stained with crystal violet, and the cell-covered area of visible colonies was calculated. Cell viability and cell proliferation were determined by 3-(4,5-dimethylthiazol-2-yl)-2,5-diphenyltetrazolium bromide (MTT) and EdU cell proliferation assays, respectively. For the MTT assay, 1000 cells in 200 µL culture medium were seeded into each well of 96-well plates. After cultivation, MTT was added into each well. Optical density (OD) was measured and cell growth curves were drawn according to the OD value. For the EdU cell proliferation assay, as described previously (51), EdU DNA Cell Proliferation kit (RiboBio Co. Ltd., Guangzhou, China) was used (52). Briefly, cells (1×10^5^) were cultured in 24-well plates, and then, exposed to 50 µM EdU for 2 h at 37°C. The cells were fixed with 4% formaldehyde and permeabilized in 0.5% Triton X-100. Cells were then incubated with 200 µM Apollo reaction cocktail, and nuclei were stained with Hoechst 33342 (200 mL/well). The stained cells were visualized under a fluorescence microscope (IX71; Olympus, Tokyo, Japan). The number of cells was counted using Image-Pro Plus 6.2 software (Meyer Instruments, Inc., Houston, TX, USA).

### Cell Migration Assay

Migration of cells was evaluated by wound-healing assay *via* uncoated Transwell^®^ cell culture inserts according to the manufacturer’s instructions (Corning Inc., New York, NY, USA). To carry out wound-healing assay, monolayer of cells was gently scratched with sterile micropipette tips, washed with phosphate-buffered saline (PBS), and incubated in a FBS-free medium with mitomycin. The areas of the gap at 0 h and the residual gap at 24 h after wounding of 10 random locations were compared. For the uncoated Transwell^®^ assay, the uncoated Transwell^®^ filter inserts with 8-µm pores (BD Biosciences, Bedford, MA, USA) in 24-well cell culture plates were used. Then, 1 × 10^5^ cells were suspended and seeded into the uncoated and precoated upper chambers of 24-well Transwell^®^ plates with a FBS-free medium. The medium containing 10% FBS was added to the lower chamber to serve as a chemo-attractant. After 12 h of incubation, migrated cells were stained with 0.5% crystal violet solution. Cell migration was quantified by calculating the cell-covered area in photomicrographs using Image-Pro Plus v6.2 software.

### Cell Apoptosis Assay

For cell apoptosis analysis, cells were cultured in FBS-free medium for 48 h, followed by staining with the Annexin V-fluorescein isothiocyanate (FITC)/7-amino-actinomycin D (7-AAD) (BD Biosciences, San Diego, CA, USA). For each assay, 1 × 10^5^ cells were incubated with Annexin V-FITC/7-AAD. After addition of 400 μL binding buffer, the cells were analyzed by fluorescence-activated cell sorting using FACScan (BD Biosciences, San Diego, CA, USA). Cell populations were classified as viable (Annexin V negative, 7-AAD negative), apoptotic (Annexin V positive, 7-AAD negative or positive), or necrotic (Annexin V negative, 7-AAD positive).

### Xenograft Mouse Model

Athymic nude mice (strain BALB/c nu/nu; Charles River Laboratories, Wilmington, MA, USA) were used for tumorigenesis studies. These mice (age, 8-12-week-old) were housed and maintained under specific pathogen-free conditions in facilities approved by the Hubei Provincial Association for Laboratory Animal Sciences (China). For the subcutaneous xenograft mouse model, HCT116 cells (Lenti-Nkx2.5-HCT116 and Lenti-NC-HCT116, 1×10^6^ cells/well) were harvested and resuspended in Hanks’ Balanced Salt Solution. These two HCT116 cells were subcutaneously injected at day 0 into the right and left dorsal flanks, respectively. Tumor diameter was measured every 2 days before harvesting over the course of 32 days. Tumor volume (mm^3^) was calculated by measuring the shortest (x) and longest (y) diameters of the tumor using the following formula: tumor volume (mm^3^) = x^2^y/2.

### Terminal Deoxynucleotidyl Transferase-Mediated dUTP Nick-End Labeling (TUNEL) Assay

In-situ cell death was determined by TUNEL staining of tumor tissue sections (In Situ Cell Death Detection Kit, cat. no. 11684817910; Roche Applied Science, Penzberg, Germany). Staining was performed in strict accordance with the manufacturer’s instructions. Briefly, frozen slides were dried and fixed with 4% paraformaldehyde. The Proteinase K solution and 1% Triton X-100 were added. TdT (terminal deoxynucleotidyl transferase), fluorescein-isothiocyanate (FITC)-labeled d-UTP mixed solution, and DAPI (4’,6-diamidino-2-phenylindole) were added for labeling and staining. After washing with PBS, anti-fluorescence quenching mounting medium was added for sealing. The slides were observed and photographed under a fluorescence microscope (IX71; Olympus, Tokyo, Japan). The cells with green fluorescence were apoptosis-positive cells. TUNEL-positive cells were counted using Image-Pro Plus v6.2 software in 10 randomly selected fields from each slide.

### Immunohistochemistry (IHC)

The fraction of proliferative cells in the xenograft tumor section was assessed with Ki-67 staining. Paraffin-embedded tissue sections were deparaffinized in xylene and rehydrated through graded ethanol solutions. Sections were boiled in antigen retrieval solution (EnVision FLEX Target Retrieval Solution; Agilent Technologies, Inc., Santa Clara, CA, USA) for antigen retrieval. Afterwards, endogenous peroxidases were inactivated by immersing in 3% hydrogen peroxide. The slides were blocked with 10% bovine serum albumin (BSA), incubated with the anti-Ki-67 antibody (GA62661-2; Agilent Technologies, Inc., Santa Clara, CA, USA) overnight, and then, with the corresponding secondary antibody for 1 h at room temperature. Finally, the sections were developed with DAB color solution. Sections were counterstained with hematoxylin and preserved with resinene. The cells with brown-stain were proliferation-positive cells. Ki-67-positive cells were counted using Image-Pro Plus v6.2 software in 10 randomly selected fields from each slide.

### TP53 Gene Sequencing and Mutation Analysis

Total genomic DNA was extracted from each cell line (FHC, DLD-1, HCT116, HT29, SW480). Eight pairs of primers (see **Supplementary Table 4**) were used to amplify the exonic regions of TP53. PCR amplification was performed using MyGene™ Series Peltier Thermal Cycler (A300; Hangzhou LongGene^®^ Scientific Instruments Co., Ltd., Zhejiang, China) and PrimeSTAR GXL DNA Polymerase (cat. no. R050Q; Takara Biotechnology, Inc., Otsu, Japan). DNA sequencing was carried out by using an 3730xl DNA Analyzer (Applied Biosystem, Foster City, CA, USA). All fragments were sequenced from both strands. DNA sequencing analysis was undertaken by using Chromas software (Technelysium Pty Ltd., Queensland, Australia).

### Co-immunoprecipitation (Co-IP) Assay

Co-IP was conducted *via* a Co-IP kit (Pierce^™^ Co-Immunoprecipitation-Kit, No. 26149; Thermo Fisher Scientific, Waltham, MA, USA), according to the manufacturer’s instructions as previously reported (53, 54). Briefly, anti-Nkx2.5 antibody (sc-376565X; Santa Cruz Biotechnology, Dallas, TX, USA) was immobilized by incubating with AminoLink plus coupling resin in the Pierce spin columns to prepare anti-Nkx2.5-coated resin, and control mouse IgG antibody (sc-2025; Santa Cruz Biotechnology, Dallas, TX, USA) was used to prepare IgG-coated resin with the same procedure. Total cell protein extracts of HCT116 cells (Lenti-NC-HCT116 and Lenti-Nkx2.5-HCT116) were prepared using ice-cold IP lysis buffer. Extracts were added to the Pierce spin columns with antibody-coated resins, resulting in making four combinations (Lenti-NC-HCT116 + IgG, Lenti-NC-HCT116 + Anti-Nkx2.5, Lenti-Nkx2.5-HCT116 + IgG, and Lenti-Nkx2.5-HCT116 + Anti-Nkx2.5). After incubation and elution, the eluates containing the protein complexes were collected. Finally, aliquots were denatured by heating at 100°C and analyzed by SDS-PAGE and Western blotting.

### Luciferase Reporter Assay

For luciferase reporter vector construction, the pGL3.0 p21^WAF1/CIP1^ luciferase reporter vector (p21^WAF1/CIP1^-Luc) was provided by Promega (Madison, WI, USA). Luciferase activity assay was undertaken as previously described (55, 56). In brief, the reporter vectors were transfected into HCT116 cells (Lenti-NC-HCT116 and Lenti-Nkx2.5-HCT116) using Lipofectamine 2000 reagent (Invitrogen, Carlsbad, CA, USA). Then, pRL-SV40 vector expressing Renilla luciferase was co-transfected as an internal control to normalize transfection efficiency. Luciferase activities were measured 24 h after transfection. The relative luciferase activity was calculated as luciferase activity of reporter vectors in HCT116 cells (Lenti-NC-HCT116 and Lenti-Nkx2.5-HCT116).

### Statistical Analysis

The statistical analysis was performed by using GraphPad Prism 8.4 software (GraphPad Software Inc., La Jolla, CA, USA). All data were expressed as mean ± standard deviation (SD). The significant differences were analyzed by the log-rank test for KM survival analysis, Wilcoxon matched-pairs signed-rank test and paired t-test for comparing paired data between two groups, Mann-Whitney U test and unpaired t-test with Welch’s correction for comparing unpaired data between two groups, and Mann-Whitney U test or Brown-Forsythe and Welch’s analysis of variance (ANOVA) with Dunnett’s post-hoc test for making multiple comparisons. P < 0.05 was considered statistically significant.

## Results

### Nkx2.5 Is Highly Expressed in CRC Samples

**Figure 1:**
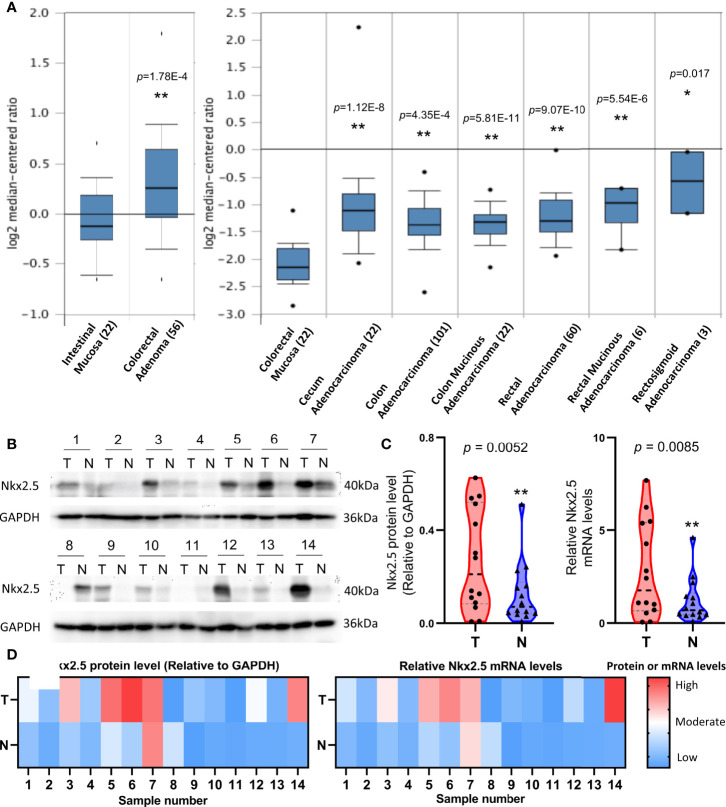
Higher expression level of Nkx2.5 in CRC samples than that in NCM samples. **(A)** Expression level of Nkx2.5 in colorectal adenomas, CRC, and NCM tissues. The graph was downloaded from ONCOMINE database. The numbers in brackets represent the sample size. **(B)** Western blot analysis was employed to measure Nkx2.5 protein level in 14 paired CRC and NCM samples. **(C)** RT-qPCR was used to detect Nkx2.5 mRNA level in CRC and NCM samples. **(D)** Protein and mRNA levels of Nkx2.5 in 14 paired CRC and NCM samples presented by heat maps. “T” represents CRC sample; “N” represents NCM sample. * represents *P* < 0.05; ** represents *P* < 0.01; ns represents *P* ≥ 0.05 (no statistical significance).

To indicate whether Nkx2.5 expression differs between CRC and NCM samples, microarray data that were extracted from ONCOMINE database were analyzed. A significantly higher expression of Nkx2.5 was detected in colorectal adenoma (Gaspar Colon dataset) and CRC tissue (TCGA Colorectal dataset) than that in NCM tissue (**Figure 1A**). To verify the results from database, Nkx2.5 profiles from 14 paired CRC and NCM samples (**Supplementary Table 1**) were examined by Western blotting and RT-qPCR. Nkx2.5 protein level (*P* = 0.0052 for Wilcoxon matched-pairs signed-rank test, *P* = 0.0139 for paired t-test) and mRNA expression (*P* = 0.0085 for Wilcoxon matched-pairs signed-rank test, *P* = 0.0264 for paired t-test) were observed to be significantly higher in CRC samples than those in NCM samples (**Figures 1B–D**).

### High Expression of Nkx2.5 Is Correlated With a Poor Differentiation of CRC Cells

**Figure 2:**
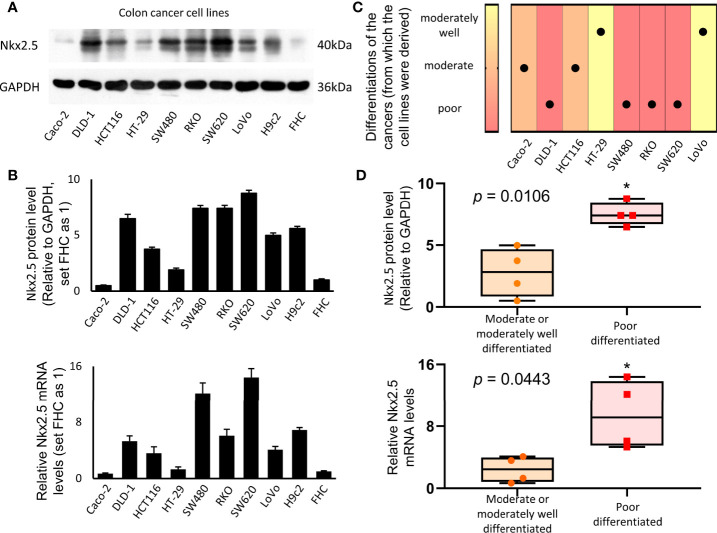
Higher expression of Nkx2.5 in poorly-differentiated CRC cell lines. **(A)** Western blotting was used to identify the expression of Nkx2.5 in CRC cell lines (H9c2 and FHC serve as positive and negative controls, respectively). **(B)** RT-qPCR was used to detect Nkx2.5 mRNA level in CRC cell lines. **(C)** Pathological differentiation degrees of the original CRC tissues. **(D)** Nkx2.5 protein and mRNA levels in the moderately- or well-differentiated CRC cell lines compared with poorly-differentiated CRC cell lines. The upper and lower bars indicate the maximum and minimum values, respectively. * represents *P* < 0.05.

Protein and mRNA levels of Nkx2.5 in a panel of eight CRC cell lines (Caco-2, DLD-1, HCT116, HT-29, SW480, RKO, SW620, and LoVo), one normal colon epithelial cell (FHC), and one rat cardiomyocyte (H9c2, Nkx2.5-positive cell) were analyzed. Protein and mRNA levels of Nkx2.5 were high in H9c2, low in FHC, and diversely expressed among CRC cell lines (**Figures 2A, B**). We analyzed the correlation between histological grades (49) of the original tumors (**Figure 2C**) (57–59) and Nkx2.5 levels in the CRC cell lines (**Figure 2B**). It was observed that higher Nkx2.5 protein (*P* = 0.0286 for Mann-Whitney U test, *P* = 0.0051 for unpaired t-test with Welch’s correction; **Figure 2D**) and mRNA (*P* = 0.0286 for Mann-Whitney U test, *P* = 0.0443 for unpaired t-test with Welch’s correction; **Figure 2D**) levels significantly correlated with higher histological grades.

### Nkx2.5 Suppresses Proliferation, Promote Apoptosis, While It Does Not Affect Migration of HCT116 Cells *In Vitro*

**Figure 3:**
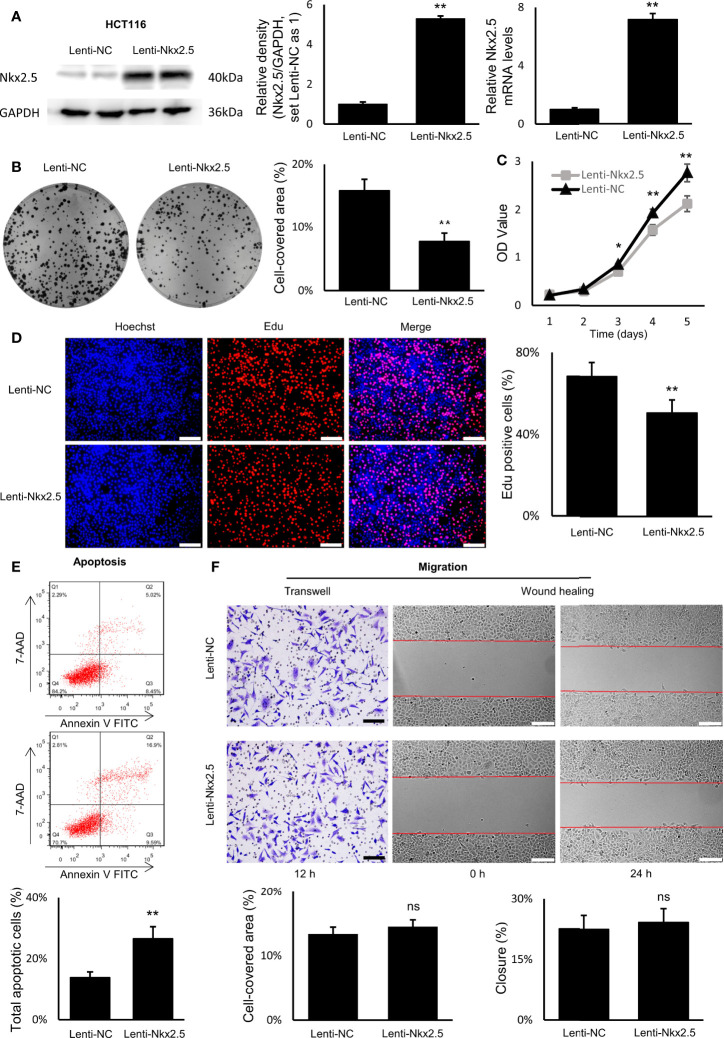
Nkx2.5 could suppress proliferation and induce apoptosis in cultured HCT116 cells. **(A)** Detection of the protein and mRNA levels of Nkx2.5 in Lenti-NC and Lenti-Nkx2.5 HCT116 cells by Western blotting and RT-qPCR. GAPDH was used as an endogenous control gene. **(B)** Representative photographs of colony formation of the Lenti-NC and Lenti-Nkx2.5 HCT116 cells. Cell-covered area of colonies was quantified and shown as percentage (%) relative to area of the well bottom. **(C)** Cell growth of the Lenti-NC and Lenti-Nkx2.5 HCT116 cells was determined by MTT assay at each time-point. **(D)** Representative profiles of EdU cell proliferation assay in the Lenti-NC and Lenti-Nkx2.5 HCT116 cells. Representative photographs were taken at magnification of 200×. Scale bar (white) is 100-μm. **(E)** Apoptosis of Lenti-NC and Lenti-Nkx2.5 HCT116 cells was measured by flow cytometry. Representative histograms show cell population in apoptotic (top right and bottom right quadrants), viable (bottom left quadrant), and necrotic (top left quadrant) states. **(F)** Cell migration was assessed by Transwell^®^ assay and wound-healing assay. Representative photographs were taken at magnifications of 200× and 100× for Transwell^®^ assay and wound-healing assay, respectively. Scale bar (black) is 100-μm. Scale bar (white) is 200-μm. * represents *P* < 0.05; ** represents *P* < 0.01; ns represents *P* ≥ 0.05 (no statistical significance).

To explore the function of Nkx2.5, Nkx2.5-overexpressed cells (Lenti-Nkx2.5-HCT116) were established using a lentiviral vector in HCT116 cells. Being derived from a moderately poor differentiated CRC cancer, HCT116 cell line showed to have a moderate proliferation rate and apoptosis rate among CRC cell lines (57–59). Thus, HCT116 could be an appropriate cellular model to show how the proliferation and/or apoptosis was affected (60). Moreover, Nkx2.5 was moderately expressed in HCT116 cells (**Figures 2A, B**), thus overexpression of Nkx2.5 in HCT116 cells may result in a positive phenotype. HCT116 with Nkx2.5 overexpression showed significantly higher Nkx2.5 protein and mRNA levels (**Figure 3A**; Mann-Whitney U test: *P* < 0.01; unpaired t-test with Welch’s correction: *P* < 0.0001) than those in the control cells with an empty vector (Lenti-NC-HCT116).

In colony formation assay, Nkx2.5 overexpression dramatically reduced the colony formation efficiency in HCT116 cells (**Figure 3B**, Mann-Whitney U test: *P* < 0.01 and unpaired t-test with Welch’s correction: *P* < 0.0001). In MTT and EdU proliferation assay, Nkx2.5 overexpression significantly inhibited cell growth and decreased proliferation rate in HCT116 cells (**Figures 3C, D**, Mann-Whitney U test: *P* < 0.0001 and unpaired t-test with Welch’s correction: *P* < 0.0001). These results suggest that Nkx2.5 expression contributed to the reduced growth or proliferation in HCT116 cells. Additionally, flow cytometry revealed a remarkably increased apoptotic cell fraction in Nkx2.5 overexpressed cells (26.57 ± 3.91% versus 13.84 ± 1.80%; **Figure 3E**, Mann-Whitney U test: *P* < 0.01 and unpaired t-test with Welch’s correction: *P* < 0.001). In wound-healing and Transwell assays, Nkx2.5 overexpression did not affect HCT116 cell migration. (**Figure 3F**).

Taken together, the above-mentioned findings demonstrate that Nkx2.5 plays a significant role in suppressing proliferation and promoting apoptosis of HCT116 cells *in vitro*. It may also be noted that migration of HCT116 cells was not influenced by Nkx2.5 overexpression.

### Nkx2.5 Suppresses Formation and Growth of Tumor Cells and Promotes Apoptosis of CRC Cells *In Vivo*

**Figure 4:**
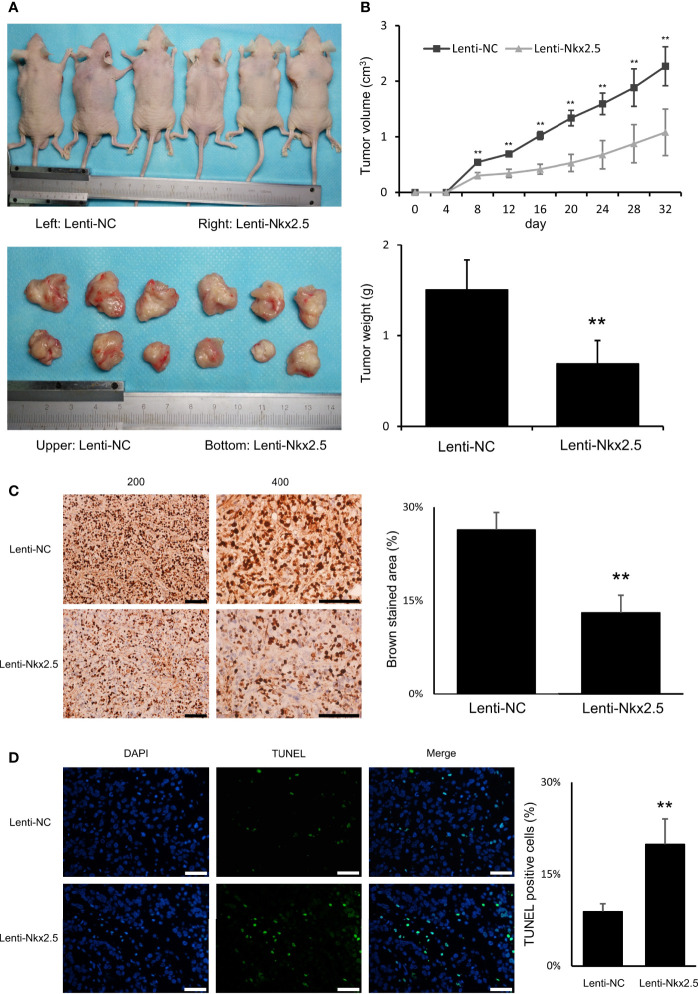
Nkx2.5 could suppress tumor formation, growth, proliferation, and increase apoptosis of HCT116 cells *in vivo*. **(A)** Photographs of tumors in mice that were excised 32 days after inoculation of Lenti-NC and Lenti-Nkx2.5 HCT116 cells into athymic nude mice. **(B)** Growth of tumor volume was plotted over time, and weight of tumor at the end of 32 days was illustrated (6 mice/group). **(C)** Immunohistochemical staining of Lenti-NC and Lenti-Nkx2.5 cells using Ki-67. Representative photographs were taken at magnifications of 200× and 400×. Scale bar (black) is 100-μm. **(D)** Representative profiles for TUNEL assay of the Lenti-NC and Lenti-Nkx2.5 cells. Green fluorescence indicates TUNEL-positive cells in the microscopic field. DAPI was used for nuclear staining. Representative photographs were taken at magnification of 400×. Scale bar (white) is 50-μm. ** represents *P* < 0.01.

For *in vivo* assay, Lenti-Nkx2.5-HCT116 and Lenti-NC-HCT116 cells were subcutaneously injected into nude mice. Nkx2.5 overexpression remarkably inhibited formation and growth of tumor cells *in vivo* (*P* < 0.01 for Mann-Whitney U test; **Figure 4A**), according to the measurement of the volume and weight of xenograft tumors (*P* < 0.01 for Mann-Whitney U test; **Figure 4B**). Moreover, Ki-67 staining and TUNEL staining showed a lower proliferation rate (*P* < 0.01 for Mann-Whitney U test; **Figure 4C**) with a higher apoptosis rate (*P* < 0.01 for Mann-Whitney U test; **Figure 4D**) in Nkx2.5 overexpressed xenograft tumors. These findings suggest that Nkx2.5 functions as a tumor suppressor in HCT116 cells *in vivo*.

### Nkx2.5 Overexpression Does Not Affect Proliferation, Apoptosis, and Migration of SW480 Cells *In Vitro*

**Figure 5:**
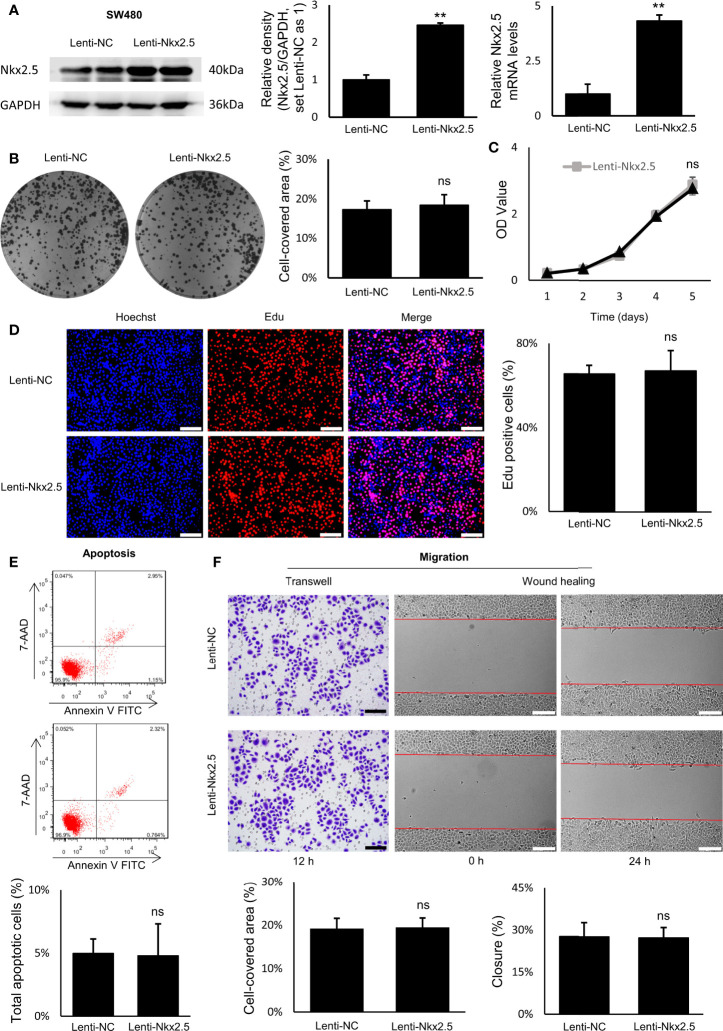
Proliferation, apoptosis, and migration were not affected by Nkx2.5 overexpression in SW480 cells. **(A)** Detection of the protein and mRNA levels of Nkx2.5 in the Lenti-NC and Lenti-Nkx2.5 SW480 cells by Western blotting and RT-qPCR. GAPDH was used as an endogenous control gene. **(B)** Representative photographs of colony formation of the Lenti-NC and Lenti-Nkx2.5 SW480 cells. Cell-covered area of colonies was quantified and shown as percentage (%) relative to area of the well bottom. **(C)** Cell growth of the Lenti-NC and Lenti-Nkx2.5 SW480 cells was determined by MTT assay at each time-point. **(D)** Representative profiles for EdU cell proliferation assay of the Lenti-NC and Lenti-Nkx2.5 SW480 cells. Representative photographs were taken at magnification of 200×. Scale bar (white) is 100-μm. **(E)** Apoptosis of Lenti-NC and Lenti-Nkx2.5 SW480 cells was measured by flow cytometry. Representative histograms show cell population in apoptotic (top right and bottom right quadrants), viable (bottom left quadrant), and necrotic (top left quadrant) states. **(F)** Cell migration was assessed by Transwell^®^ assay and wound-healing assay. Representative photographs were taken at magnifications of 200× and 100× for Transwell^®^ assay and wound-healing assay, respectively. Scale bar (black) is 100-μm. Scale bar (white) is 200-μm. ** represents *P* < 0.01; ns represents *P* ≥ 0.05 (no statistical significance).

In order to confirm the role of Nkx2.5 in CRC cells, studies were also carried out in a second cell line SW480 which is derived from a poorly differentiated CRC having high proliferation rate and a low apoptosis rate among CRC cell lines (57–59). It is therefore highly appropriate for testing of the tumor suppressive effect of a gene or a drug. Moreover, in contrast to the low expression of Nkx2.5 in HCT116 cells, Nkx2.5 was highly expressed in SW480 cells. Thus, we attempted to assess whether a higher Nkx2.5 expression could suppress SW480 cells. Then, Nkx2.5-overexpressed and the control SW480 cells (Lenti-NC-SW480 and Lenti-Nkx2.5-SW480) were established. The transfection efficiency was confirmed by Western blotting and RT-qPCR (**Figure 5A**, *P* < 0.01).

Overexpression of Nkx2.5 in SW480 cells did not influence neither the efficiency of colony formation (**Figure 5B**) nor cell growth evaluated by MTT assay (**Figure 5C**). EdU proliferation assay showed no significant difference in proliferation rate between Lenti-NC-SW480 and Lenti-Nkx2.5-SW480 cells (**Figure 5D**). In addition, flow cytometry showed no significant difference in apoptosis rate between Lenti-NC-SW480 and Lenti-Nkx2.5-SW480 cells (4.99 ± 1.13% versus 4.80 ± 2.52%; **Figure 5E**). These outcomes suggest that overexpression of Nkx2.5 did not affect proliferation or apoptosis of SW480 cells. In wound-healing and Transwell assays, the migration of SW480 cells also could not be affected by Nkx2.5 overexpression (**Figure 5F**).

In contrast to the results in HCT116 cells, these results suggest that Nkx2.5 overexpression could not affect proliferation, apoptosis, or migration of SW480 cells.

### Protein Levels of p53 and p21 Are Varied in Different CRC Cell Lines

**Figure 6:**
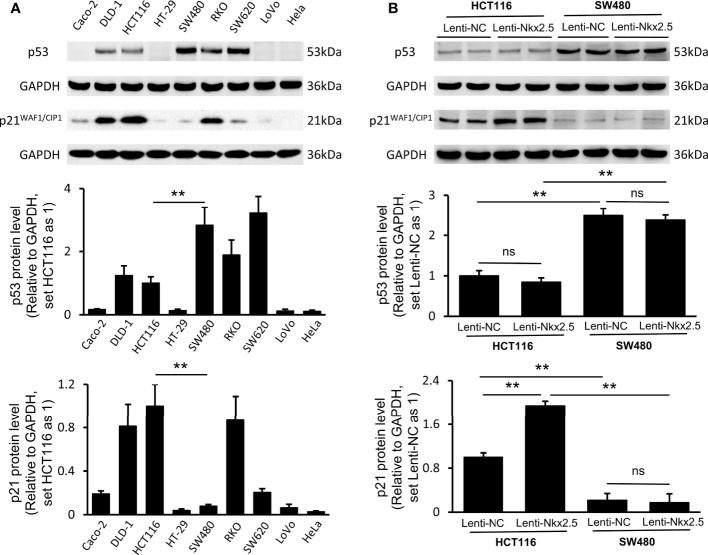
Expression of p53 and p21^WAF1/CIP1^ in CRC cell lines and Nkx2.5 overexpressed CRC cells. **(A)** Western blotting and RT-qPCR were employed for detecting protein levels of p53 and p21^WAF1/CIP1^ in CRC cell lines. **(B)** Western blotting and RT-qPCR were used for detecting mRNA levels of p53 and p21^WAF1/CIP1^ in Lenti-NC and Lenti-Nkx2.5 CRC cells. GAPDH was used as an endogenous control gene. ** represents *P* < 0.01; ns represents *P* ≥ 0.05 (no statistical significance).

In order to determine why different CRC cell lines exhibit different patterns when Nkx2.5 is overexpressed, we measured the levels of antitumor protein p53 (38, 40, 61–63) and its downstream effector p21^WAF1/CIP1^ (64, 65) since Nkx2.5 behaves like p53 and it may interact with p53-related pathway as another NK2 family member Nkx2.1 (17–19, 25). Western blotting showed that p53 and p21^WAF1/CIP1^ were diversely expressed in CRC cell lines (*P* < 0.001 for Brown-Forsythe and Welch’s ANOVA with Dunnett’s post-hoc test), while Hela cell (66) served as a negative control (**Figure 6A**). Strikingly, HCT116 cells showed a moderately lower level of p53, whereas a relatively higher level of p21^WAF1/CIP1^ was observed in HCT116 cells compared with that in SW480 cells (*P* < 0.01 for Mann-Whitney U test; **Figure 6A**).

Lenti-Nkx2.5-HCT116 cells possessed the same level of p53, while a higher level of p21^WAF1/CIP1^ than that in Lenti-NC-HCT116 cells (*P* < 0.01 for Mann-Whitney U test; **Figure 6B**). Lenti-NC-SW480 and Lenti-Nkx2.5-SW480 cells had the same levels of p53 and p21^WAF1/CIP1^ (**Figure 6B**). This result suggests that Nkx2.5 increased p21^WAF1/CIP1^ expression in HCT116 but not in SW480. The p53 level in Lenti-NC-HCT116 and Lenti-Nkx2.5-HCT116 cells was remarkably lower than that in Lenti-NC-SW480 and Lenti-Nkx2.5-SW480 cells, while p21^WAF1/CIP1^ level in Lenti-NC-HCT116 and Lenti-Nkx2.5-HCT116 cells was significantly higher than that in Lenti-NC-SW480 and Lenti-Nkx2.5-SW480 cells (*P* < 0.01 for Mann-Whitney U test; **Figure 6B**). This result suggests that p53 loses its transcriptional activity for p21^WAF1/CIP1^ in SW480 but not in HCT116, even though SW480 cells possess high level of p53.

Put together, these results indicate that Nkx2.5 overexpression can increase p21^WAF1/CIP1^ expression without influencing p53 level in HCT116 cells, while p53 seems to lose its function in SW480 cells.

### Levels of Nkx2.5 and p21^WAF1/CIP1^ Are Correlated to the Mutational Status and Protein Level of p53

**Figure 7:**
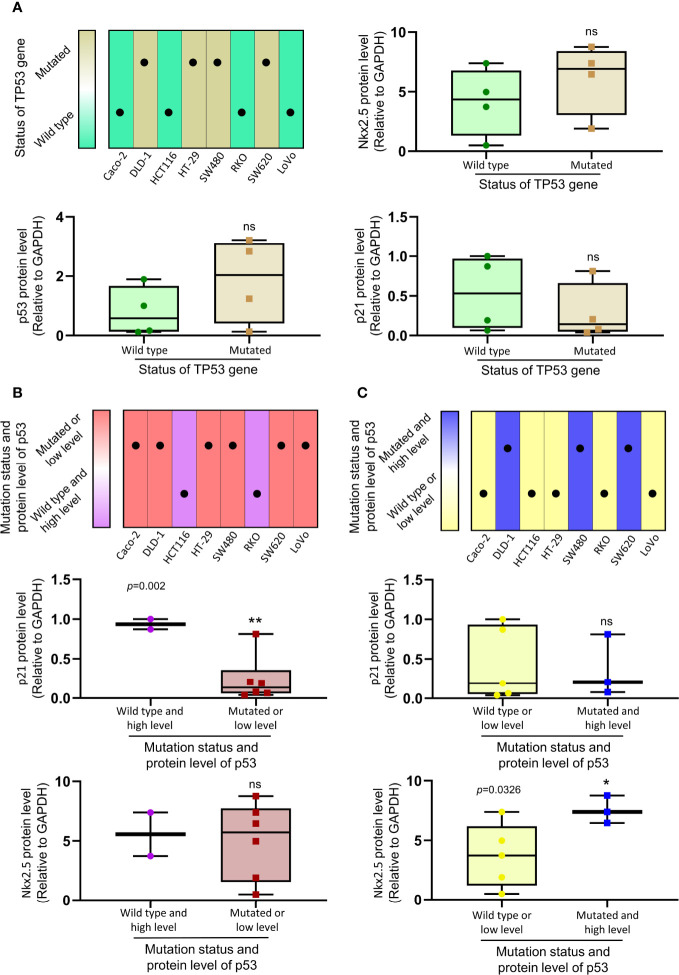
The relationship between the expression of Nkx2.5, p21^WAF1/CIP1^, p53 and mutational status of TP53. **(A)** Protein levels of Nkx2.5, p53, and p21^WAF1/CIP1^ were compared between CRC cell lines with wild-type TP53 and those with mutated TP53. **(B)** Protein levels of p21^WAF1/CIP1^ and Nkx2.5 were compared between CRC cell lines with high expression of wild-type p53 and those with mutated or low expression of wild-type p53. **(C)** Protein levels of p21^WAF1/CIP1^ and Nkx2.5 were compared between CRC cell lines with high expression of mutated p53 and those with wild-type or low expression of mutated p53. * represents *P* < 0.05; ** represents *P* < 0.01; ns represents *P* ≥ 0.05 (no statistical significance).

**Figure 8:**
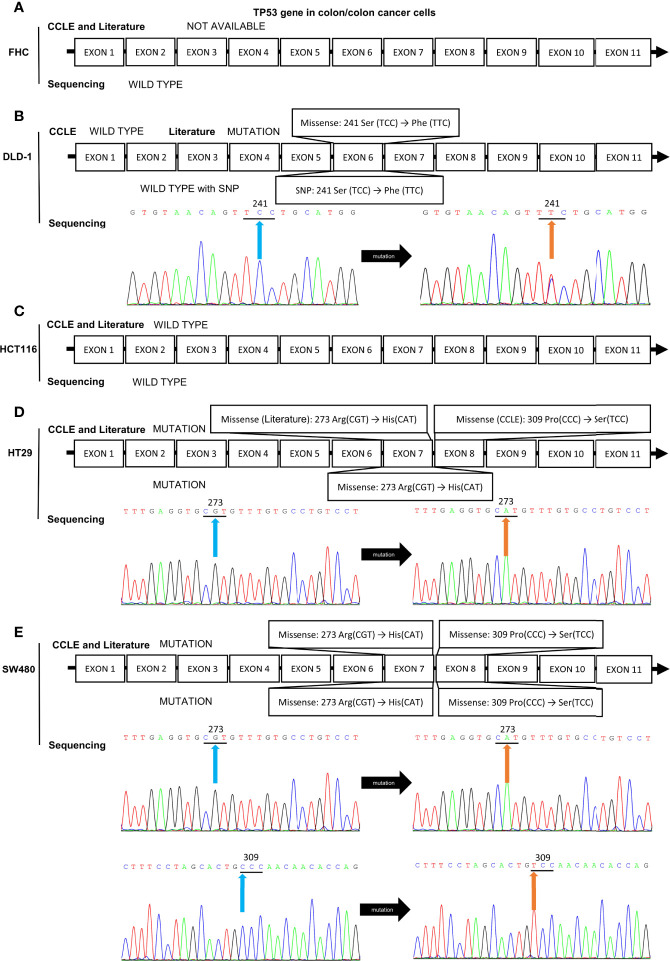
Mutation analysis of the TP53 gene in CRC cell lines by reviewing literatures, retrieving data in CCLE database, and DNA sequencing analysis. The mutational status of TP53 gene in **(A)** FHC, **(B)** DLD-1, **(C)** HCT116, **(D)** HT-29, and **(E)** SW480 cell lines. The blue and orange arrows indicate the locations of the mutation sites.

Because of the abnormal protein level of p53/p21^WAF1/CIP1^ in SW480 and SW620 cells, the mutational status of TP53 in CRC cell lines was assessed as in previous studies (57, 67) and the CCLE database (https://xenabrowser.net/heatmap/) (**Figure 7A**). DNA sequencing analysis was also conducted to confirm the mutational status of TP53 in CRC cell lines (**Figure 8**).

To investigate the relationship between the levels of Nkx2.5, p53, and p21^WAF1/CIP1^, eight CRC cell lines were assigned to two groups (wild-type p53 versus mutated p53; **Figure 7A**). No significant differences in the levels of Nkx2.5, p53, and p21^WAF1/CIP1^ were observed between these two groups. Since either mutational (38) or transcriptional (40) inactivation of TP53 could lead to functional disruption of p53, the CRC cell lines were allocated to two groups in another way (high expression of wild-type p53 versus mutated or low expression of wild-type p53, median level was chosen as cutoff value; **Figure 7B**). A significantly higher expression of p21^WAF1/CIP1^ was observed in the former group (high expression of wild-type p53: HCT116 and RKO cells). Since mutated p53 was highly expressed in high malignancies and reported as an oncogene gaining new activities (68, 69), we assigned the CRC cell lines to two groups (wild-type or low expression of mutated p53 versus high expression of mutated p53, median level was chosen as cutoff value; **Figure 7C**). A markedly higher expression of Nkx2.5 was noted in the latter group (high expression of mutated p53: DLD-1, SW480, and SW620 cells).

### Nkx2.5 Can Interact With p53 and Increase Its Transcriptional Activity

**Figure 9:**
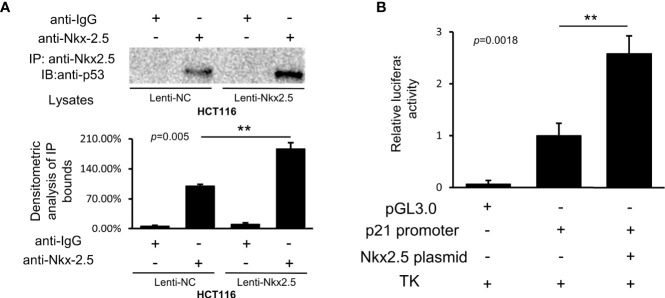
Nkx2.5 interacts with p53 and activates the transcriptional activity of p53. **(A)** Image of immunocomplexes from Lenti-NC and Lenti-Nkx2.5 HCT116 cells that were blotted with anti-p53 monoclonal antibody. **(B)** Luciferase reporter assay revealed that Nkx2.5 activated the p21WAF1/CIP1 promoter in HCT116 cells. ** represents *P* < 0.01; ns represents *P* ≥ 0.05 (no statistical significance).

Co-IP assay showed that Nkx2.5 could interact and co-precipitate with p53 in HCT116 cells (**Figure 9A**). The luciferase activity assay showed an additive effect on the activity of the p21^WAF1/CIP1^ promoter by Nkx2.5 plasmid (**Figure 9B**). The results highlight that Nkx2.5 can increase p21^WAF1/CIP1^ expression by interacting with p53 as a coactivator.

## Discussion

Nkx2.5 was considered as a cardiac specific transcription regulator since it is preferentially expressed in heart (12, 70–74). To date, very little has been known about the roles of Nkx2.5 in other organs or tumors. Recent reports about the expression of Nkx2.5 in several types of tumors (27–30) implied its potential role in tumors, but the underlying mechanism has not been clearly elucidated. The findings in our present study indicates that Nkx2.5 serves as a conditional tumor suppressor in CRC cells. And its tumor suppressive effect depends on the mutational status of p53.

The first clue about Nkx2.5 in CRC cells was obtained from previously reported results, which indicated hypermethylation status of a tumor suppressor gene in a CRC cell line and several CRC samples (10). Then, this observation was confirmed in further CRC cell lines and samples (9). Thus, Nkx2.5 was considered as a candidate tumor suppressor gene for CRC (9, 10). Based on the above-mentioned outcomes, we analyzed the data of Nkx2.5 expression collected from different databases to indicate whether the expression level of Nkx2.5 could be downregulated in CRC tissues because of hypermethylation. However, the expression level of Nkx2.5 in colorectal adenoma and CRC samples was significantly higher than that in NCM samples (**Figure 1A**), which is not consistent with our expectation. Results acquired from other datasets showed that the expression of Nkx2.5 in CRC and NCM samples was not significantly different from each other (data not shown). However, no result could be achieved regarding higher expression of Nkx2.5 in NCM samples than that in CRC samples, indicating that a portion of CRCs possessed high level of Nkx2.5, while others possessed normal level of Nkx2.5 as that in NCM samples. However, non-paired data is a big limitation of data analysis results from databases. We therefore attempted to obtain some more paired data from CRC patients. Then, 14 paired CRC and NCM samples were collected and analyzed. Higher protein and mRNA levels of Nkx2.5 were detected in CRC samples (**Figures 1B–D**), which is consistent with the findings from databases (**Figure 1A**). Nkx2.5 has also been observed to be highly expressed in squamous cell carcinoma of skin (28), T-cell acute lymphoblastic leukemia (30), ovarian yolk sac tumor (29), and papillary thyroid carcinoma (27), while lowly expressed in basal cell carcinoma of skin (28). These studies indicate the diversity of Nkx2.5 expression in tumors, but its role in tumor is still unclear.

Different CRC cell lines were used as models for different histological grades of CRC. After classification of the CRC cell lines into three grades according to the histological grades of the original tumors (**Figure 2C**), significantly higher protein and mRNA levels of Nkx2.5 were observed in poorly-differentiated CRC cell lines than those in moderately- or well-differentiated CRC cell lines (**Figure 2D**). Taking the results achieved from databases, CRC samples and CRC cell lines together, we found that Nkx2.5 is highly expressed in both CRC tissue and cell lines, especially in poorly differentiated cell lines. These results imply that Nkx2.5 behaves like an oncogene rather than a tumor suppressor gene. But the current study originally aimed to verify the tumor suppressive role of Nkx2.5 in CRC.

To investigate the role of Nkx2.5 in CRC cells, Nkx2.5 overexpressed HCT116 and SW480 cell lines were established to indicate how Nkx2.5 could affect CRC cells. As illustrated in **Figures 3**, **4**, Nkx2.5 could act as a tumor suppressor gene in HCT116 cells *in vitro* and *in vivo*, while it was incapacitated in SW480 cells (**Figure 5**). These results suggest that Nkx2.5 may serve as a tumor suppressor in certain CRC cells.

On one hand, Nkx2.5 was highly expressed in CRC cells, especially highly malignant cell lines, as an oncogene. On the other hand, Nkx2.5 was noted as a tumor suppressor in a certain CRC cell line. This contradiction reminds us of TP53, a well-accepted tumor suppressor gene encoding p53 protein (36, 75, 76). Since numerous tumors produce abundant p53 protein than normal tissues, TP53 was considered to be an oncogene in the first decade after its discovery (77, 78). Then, the hypothesis that TP53 acts as an oncogene was overturned (79–81). It has been accepted that wild-type TP53 is a tumor suppressor gene, whereas TP53 mutants act as oncogene (68). TP53 mutants have been found in more than 50% of cancer patients (61, 82). TP53 missense mutation mainly occurs in its DNA-binding domain (38) that perturbs p53 function or disrupts the upstream or downstream regulatory networks of p53 (69). Even in those types of cancers that retain wild-type p53, the expression or function of p53 is often downregulated or inactivated (40). In the present study, we expressed wild-type Nkx2.5 in both CRC cell lines but achieved distinct results. Being inspired by the work of Kojic et al. (48), we hypothesized that Nkx2.5 may interact with p53 in CRC cells. The distinct manifestations obtained in different CRC cell lines may be attributed by the different levels or mutational status of p53 in these cells.

We noted that HCT116 cells possessed a moderately low expression of wild-type p53, while SW480 possessed high expression of mutated p53. Meanwhile, manipulation of Nkx2.5 did not influence the expression of p53 in both HCT116 and SW480 cells (**Figure 6B**). These findings indicate that the tumor suppressive effect of Nkx2.5 is not mediated by affecting p53 expression in CRC cells. Hence, we focused on its activity and downstream pathway.

P21^WAF1/CIP1^ is a broad-acting CDK inhibitor (41) which is encoded by CDKN1A gene and can interact with various cell cycle-related proteins (83–90). P53 is the major transcriptional activator of CDKN1A gene (38–40, 65, 91–94). P21^WAF1/CIP1^ was reported to be constantly diminished in p53-mutated tumor cells (95, 96). We found that p21^WAF1/CIP1^ expression was significantly higher, and Nkx2.5 overexpression could significantly elevate the expression of p21^WAF1/CIP1^ in HCT116 (wild-type p53) than SW480 (mutated p53) cells (**Figure 6B**). These results suggest that Nkx2.5 increases p21^WAF1/CIP1^ expression without affecting p53 expression in p53-wildtype CRC cells. But in p53-mutated cells, Nkx2.5 is incapable to affect the expression of either p53 or p21^WAF1/CIP1^.

To further analyze the relationship between Nkx2.5 and p53/p21^WAF1/CIP1^ pathway. It was revealed that the majority of TP53 mutated cell lines possessed low expression of p21^WAF1/CIP1^ (**Figure 6A**). However, there was no significant difference in p21^WAF1/CIP1^ expression between CRC cell lines with mutant/wild-type p53 (**Figure 7A**). We noticed that there were several cell lines with very low expression of p53 and p21^WAF1/CIP1^, even though they possess wild-type p53. Thus, we reclassified the CRC cell lines and observed significantly higher expression of p21^WAF1/CIP1^ in CRC cells with active (high expression of wild-type) p53 than the cells with inactivated (low expression or mutated) p53 (**Figure 7B**). This result indicates that the expression of p21^WAF1/CIP1^ depends on sufficient activity of p53. Meanwhile, no significant difference was noted in the expression of Nkx2.5 between these two groups (**Figure 7B**), demonstrating that Nkx2.5 should not be transcriptionally activated by wild-type p53. Since high expression of both Nkx2.5 and mutated p53 was observed in poor differentiated CRC cell lines (**Figure 2D**), and p53 mutants were reported as oncogenic proteins that lead to high malignancy (68, 69), CRC cell lines were allocated into high oncogenic p53 (high mutated p53) and low oncogenic p53 (wild-type and low mutated p53) groups. Significantly higher expression of Nkx2.5 was detected in CRC cells possessing high oncogenic p53 (**Figure 7C**). This result may explain why Nkx2.5 is highly expressed in high malignant CRC cells. Regarding he elevated Nkx2.5 in these high oncogenic CRC cell lines, it might be transcriptionally activated by mutated p53, or caused by some feedback mechanisms which is aimed to produce more p21 to control the uncontrollable proliferation of cancer cells. Though the reason why Nkx2.5 is highly expressed with mutated p53 is unknown, Nkx2.5 may serve as a biomarker for indicating the malignancy of CRC cells.

In addition, DNA sequencing analysis revealed that HCT116 and SW480 cells possessed wild-type and missense mutated p53, respectively, which is consistent with those reported in CCLE database and previous studies (57, 67, 97). HT-29 and DLD-1 cells also possessed mutated TP53 gene, while the details of mutation obtained from CCLE database, previous publications, and DNA sequencing remained inconsistent (**Figure 8**). Thus, HCT116 and SW480 could be perfect representatives for CRC cells with wild-type and mutated p53, respectively.

Since CDKN1A promoter possesses p53 binding site (98–101), rather than Nkx2.5 binding site (102), we suspected that Nkx2.5 might act as a coactivator to enhance the transcriptional activity of p53, instead of transcribing it directly. The interaction and transcriptional co-activatory effects of Nkx2.5 on p53 were confirmed by Co-IP and luciferase assays in the present study (**Figure 9**), which were also supported by another publication (48). These findings suggest that Nkx2.5 could act synergistically with p53 to activate the transcription of CDKN1A and increase the expression of p21^WAF1/CIP1^.

In summary, it was revealed that Nkx2.5 was highly expressed in CRC tissue and cell lines, relatively higher Nkx2.5 expression was observed in poorer differentiated CRC cell lines. Though the expression pattern of Nkx2.5 in tumors and cell lines makes it like an oncogene, it actually plays tumor suppressive role *via* p53/p21^WAF1/CIP1^ pathway. Nkx2.5 could not affect the expression of p53, it only interacts with and enhances the transcriptional activity of wild-type p53, resulting in increased p21 ^WAF1/CIP1^ expression and subsequent tumor suppressive effect. If p53 loses its activity because of mutation, Nkx2.5 also becomes nonfunctional. Correlation between high expression of Nkx2.5 and mutated p53 was also observed in CRC cells. Though the mechanism needs further investigations, Nkx2.5 may also serve as a biomarker for indicating the malignant degree of CRC cells. Our focus was on how Nkx2.5 interacts with wild-type p53, while the interaction with mutated p53 was not tested. There are different kinds of mutations in p53 that may endow mutant p53 with novel activities and may abrogate or sustain its interaction with Nkx2.5. It would be intricate and intriguing to investigate whether and how Nkx2.5 interacts with p53 mutants and the subsequent effects.

## Data Availability Statement

The authors acknowledge that the data presented in this study must be deposited and made publicly available in an acceptable repository, prior to publication. Frontiers cannot accept a manuscript that does not adhere to our open data policies.

## Ethics Statement

The studies involving human participants were reviewed and approved by Institutional Review Board of Union Hospital, Tongji Medical College, Huazhong University of Science and Technology (China). The patients/participants provided their written informed consent to participate in this study. The animal study was reviewed and approved by Institutional Review Board of Union Hospital, Tongji Medical College, Huazhong University of Science and Technology (China). Written informed consent was obtained from the individual(s) for the publication of any potentially identifiable images or data included in this article.

## Author Contributions

HuL, JW, KH, TZ, LG, SY, WY, and YN performed the experiments. HuL, JW, KT, LW, and KC designed the study. HoL, ZW, and GW discussed the results, presented suggestions, and made critical revisions. HuL, JW, and KH analyzed and interpreted the data. HuL, JW, and KH drafted the manuscript. KT, LW, and KC made the final approval of the version to be submitted. All authors read and approved the submitted version of the manuscript.

## Funding

This research was funded by the National Natural Science Foundation of China, grant number 81602419 to HuL and 81570568 to JW, and by the Natural Science Foundation of Hubei Province grant number 2016CFB134 to HuL. The funders had no role in the design of the study; in the collection, analyses, or interpretation of data; in the writing of the manuscript, or in the decision to publish the results.

## Conflict of Interest

The authors declare that the research was conducted in the absence of any commercial or financial relationships that could be construed as a potential conflict of interest.

## Abbreviations

ATCC, American Type Culture Collection; BCA, bicinchoninic acid; BSA, bovine serum albumin; Co-IP, co-immunoprecipitation; CRC, colorectal cancer; ECL, enhanced chemiluminescence; FBS, fetal bovine serum; GAPDH, glyceraldehyde-3-phosphate dehydrogenase; HRP, horseradish peroxidase; IHC, immunohistochemistry; KM, Kaplan-Meier; NCM, normal colonic mucosa; Nkx2.5, NK2 homeobox 5; PBS, phosphate-buffered saline; RIPA, radioimmunoprecipitation assay; RT-qPCR, quantitative reverse transcription-polymerase chain reaction; SD, standard deviation; SDS-PAGE, sodium dodecyl sulfate-polyacrylamide gel electrophoresis; STR, short tandem repeat; TCGA, The Cancer Genome Atlas.
