# Supplementary material for: Nkx2.5 Functions as a Conditional Tumor Suppressor Gene in Colorectal Cancer Cells via Acting as a Transcriptional Coactivator in p53-Mediated p21 Expression
Source: Front Oncol. 2021 Apr 1;11:648045. doi: 10.3389/fonc.2021.648045 (PMC8047315; doi:10.3389/fonc.2021.648045)
Supplement: Supplementary file 1 [file Table_1.docx]

**Supplementary Table 1** The main clinical data, including histological grades and TNM stages of 14 human subjects and TES levels of the CRC tissue samples

| **No.** | **Gender** | **Age (Years)** | **T** | **N** | **M** | **histological grade** | **Tumor TES level**  **(TES/GAPDH)** |
| --- | --- | --- | --- | --- | --- | --- | --- |
| 1  2  3  4  5  6  7  8  9  10  11  12  13  14 | Male  Female  Male  Male  Female  Female  Male  Male  Male  Male  Male  Female  Male  Female | 42  53  61  54  44  59  50  71  58  59  24  56  59  54 | 4  4  4  4  4  4  4  4  4  4  4  4  4  4 | 0  2  1  1  2  1  1  1  0  1  2  2  1  1 | 0  0  1  0  1  0  1  0  0  0  0  0  0  0 | High-grade  High-grade  High-grade  High-grade  High-grade  Low-grade  High-grade  High-grade  High-grade  High-grade  High-grade  High-grade  High-grade  High-grade | 0.281274871  0.110149318  0.426608935  0.109427875  0.546687758  0.625025778  0.537300684  0.007269391  0.12654824  0.088345295  0.008835972  0.301460312  0.068339451  0.513558938 |

**Supplementary Table 2** The Broder's grade and Duke's classification of the original tumors and TES levels of the CRC cell lines

| **Cell line** | **Duke's stage** | **Broders' grade** | **Differentiation of the original tumor** | **Histological grades** |  |
| --- | --- | --- | --- | --- | --- |
| Caco-2  DLD-1  HCT116  HT-29  SW480  RKO  SW620  LoVo | ND  C  ND  ND  B  ND  C  C | Ⅱ  Ⅲ/Ⅳ  Ⅲ/Ⅳ  Ⅰ  Ⅳ  Ⅲ/Ⅳ  Ⅳ  Ⅳ | Moderate  Poor  Moderate/ Poor  Moderately well  Poor  Poor  Poor  Moderately well | Low-grade  High-grade  High-grade  Low-grade  High-grade  High-grade  High-grade  Low-grade |  |

**ND：Not determined**

**Supplementary Table 3** The primers used for RT-qPCR

| **Gene** | **Primer Sequence (Forward and Reverse)** |
| --- | --- |
| GAPDH  p21  p53  Nkx2.5 | 5'- CCACTCCTCCACCTTTGAC -3'  5'- ACCCTGTTGCTGTAGCCA -3'  5’- TGTGGACCTGTCACTGTCTT -3’  5’- TAGGGCTTCCTCTTGGAGA -3’  5’- TTTCCGTCTGGGCTTCT -3’  5’- GCTGTGACTGCTTGTAGATGG -3’  5’- GGGATGGTAAACCGTGTCTGG -3’  5’- TAGTTGCTGTTGGACTGTGAAGG -3’ |

**Supplementary Table 4** The primers used for amplifying the exonic regions of TP53

| **Exon** | **Primer Sequence (Forward and Reverse)** |
| --- | --- |
| E1  E2/3  E4/5  E6  E7/8  E9  E10  E11 | 5'- GTTAGTATCTACGGCACCAGGTC -3'  5'- CTCTAGCCAAGCTTCCATCCCAC -3'  5’- CCACAGGAAGCCGAGCTGTCTC -3’  5’- CACTGACAGGAAGCCAAAGGGTG -3’  5’- CAATCCCAGCACTCTCAAAGAG -3’  5’- GGGTTATAGGGAGGTCAAATAAGC -3’  5’- CTCCCCTGCTTGCCACAGGTC -3’  5’- GTAGGTGGAGGAGAAGCCACAGG -3’  5'- GACAGGTAGGACCTGATTTCC -3'  5'- GGTATAAGTTGGTGTTCTGAAG -3'  5’- CTTCAGGTACTAAGTCTTGGGAC -3’  5’- GCTAGGCTAAGCTATGATGTTCC -3’  5’- GCTGTATAGGTACTTGAAGTGCAG -3’  5’- CAGGGCTGGGACCCAATGAGATGG -3’  5’- GATTTGAATTCCCGTTGTCCCAG -3’  5’- GACCCAGTCTCCAGCCTTTGTTC -3’ |
